# Resolvins: Potent Pain Inhibiting Lipid Mediators via Transient Receptor Potential Regulation

**DOI:** 10.3389/fcell.2020.584206

**Published:** 2020-12-10

**Authors:** Jueun Roh, Eun Jin Go, Jin-Woo Park, Yong Ho Kim, Chul-Kyu Park

**Affiliations:** ^1^Gachon Pain Center and Department of Physiology, College of Medicine, Gachon University, Incheon, South Korea; ^2^Department of Periodontology, School of Dentistry, Kyungpook National University, Daegu, South Korea

**Keywords:** resolvins, TRP channel, pain, inflammatory, neuropathy

## Abstract

Chronic pain is a serious condition that occurs in the peripheral nervous system (PNS) and the central nervous system (CNS). It is caused by inflammation or nerve damage that induces the release of inflammatory mediators from immune cells and/or protein kinase activation in neuronal cells. Both nervous systems are closely linked; therefore, inflammation or nerve damage in the PNS can affect the CNS (central sensitization). In this process, nociceptive transient receptor potential (TRP) channel activation and expression are increased. As a result, nociceptive neurons are activated, and pain signals to the brain are amplified and prolonged. In other words, suppressing the onset of pain signals in the PNS can suppress pain signals to the CNS. Resolvins, endogenous lipid mediators generated during the resolution phase of acute inflammation, inhibit nociceptive TRP ion channels and alleviate chronic pain. This paper summarizes the effect of resolvins in chronic pain control and discusses future scientific perspectives. Further study on the effect of resolvins on neuropathic pain will expand the scope of pain research.

## Introduction

The International Association for the Study of Pain defines pain as an unpleasant sensory and emotional experience associated with, or resembling that associated with, actual or potential tissue damage (Raja et al., 2020). Pain is classified into three types according to its cause. First, the pain generated when individuals are exposed to noxious stimuli such as extreme temperatures or sharp objects, is called nociceptive pain. Generally, pain is considered to be a negative feeling that needs to be eliminated, but nociceptive pain is protective and essential. It protects us from being injured by a hostile environment (Scholz and Woolf, 2002; Woolf, 2010). Second, inflammatory pain is caused by activation of the immune system, and it is also protective. Inflammation following tissue injury or infection is characterized by releasing proinflammatory mediators including bradykinin, prostaglandins, nerve growth factors (NGF), proinflammatory cytokines, and chemokines from immune cells. These mediators bind to pain receptors expressed in nociceptors (Woolf, 2010; Lim et al., 2015). Pathological pain is not protective and results from abnormal functioning of the nervous system. This can be categorized into two types depending on the presence of damage; one is neuropathic pain, which occurs after nerve damage; the other is dysfunctional pain, which can occur in the absence of inflammation or nerve damage (Woolf, 2010).

Inflammation and nerve damage can induce neural plasticity that results from changes in the nervous system and reduces the pain threshold. Therefore, inflammatory pain and neuropathic pain are characterized by peripheral nociceptor sensitization, arising from spontaneous nociceptor activity, hyperalgesia, and allodynia. Transient receptor potential (TRP) ion channels that are expressed in nociceptors are persistently activated by inflammatory mediators during the pathological state. TRPV1 and TRPA1 are notable nociceptive TRP ion channels, and activation of these channels can stimulate hyperexcitability of the peripheral nociceptors that transmit pain signaling to the central nervous system (CNS) (Scholz and Woolf, 2002; Patapoutian et al., 2009; Lim et al., 2015; Jardin et al., 2017; Mourot et al., 2018). Inflammatory pain includes pain from a surgical wound or an inflamed joint, while the etiology of neuropathic pain includes diabetes and chemotherapy-related peripheral neuropathy.

Resolvins (RVs) are lipid mediators that are biosynthesized from omega-3 polyunsaturated fatty acids (PUFAs) that are abundant in marine oil. Aspirin/cyclooxygenase-2 (COX-2) and other enzymes convert omega-3 docosahexaenoic acid (DHA) and eicosapentaenoic acid (EPA) to resolvin D and E through multiple mechanisms (Serhan et al., 2002). These lipid mediators are generated during the resolution phase of acute inflammation and not only terminate inflammation but also alleviate pain (Ariel and Serhan, 2007; Xu et al., 2010). The Ji lab found that the resolvin series has an analgesic effect on various types of inflammatory pain models through regulation of TRP channels (Xu et al., 2010; Park et al., 2011b).

This review will highlight TRP channels, with an emphasis on TRPV1 and TRPA1, as therapeutic targets for the resolution of both inflammatory and neuropathic pain using resolvins. To address this, pain and TRP are first described prior to resolvins. At the end, the importance of further study on the role of resolvins in neuropathic pain via TRP regulation is also suggested.

## Connectivity of Peripheral and Central Nervous Systems in Pain Pathways

### Anatomical Pain Pathway

Primary afferent neurons are pseudounipolar neurons that have one axon from the soma and bidirectional branches at the axon terminals (Amir and Devor, 2003; Gibson and Ma, 2011). The branches project to the peripheral (target organ) and central projection (spinal cord), respectively (Gibson and Ma, 2011), and transduce sensory information (mechanical, thermal, and chemical) from the peripheral to the central nervous system (Hasegawa et al., 2007).

Primary afferent nerve fibers are classified by fiber diameter or myelination and project to distinct laminae in the dorsal horn of the spinal cord. Fiber diameter and myelination are related to conduction velocity. Myelinated Aα and Aβ fibers have a large diameter and fast conduction velocity. Rapidly conducting Aβ fibers conduct proprioception and touch sensations and project to deeper laminae: III, IV, and V. However, studies have revealed that some Aβ fibers also are related to pain regulation (Xu et al., 2015; Tashima et al., 2018; Nagi et al., 2019). While thinly myelinated Aδ fibers and unmyelinated C fibers both have relatively small diameters, the former have faster conduction velocities than do the latter. Aδ fibers conduct touch, pressure, and “first” pain signals to laminae I and V. Slowly conducting C fibers respond to heat and mechanical stimuli and conduct “second” pain related signals to lamina I (peptidergic C fibers) and lamina II (non-peptidergic C fibers) (Julius and Basbaum, 2001; Furue et al., 2004; Basbaum et al., 2009). Unmyelinated C fibers and thinly myelinated Aδ fibers are activated by high-intensity stimuli (Ji and Woolf, 2001). The activation of Aδ fibers evokes sharp and pricking pain, whereas the activation of C fibers evokes a burning sensation (Bishop, 1980).

Different pathways to the brain carry different types of sensory information. Pain and heat signals from C and Aδ fibers in the superficial dorsal horn cross the midline and ascend the spinal cord. Then, sensory information is carried to the brain through the lateral spinothalamic tract (Melzack and Wall, 1962; Basbaum et al., 2009). On the other hand, proprioception and touch signals do not cross the spinal cord but ascend to the brain through the anterior spinothalamic tract (Basbaum et al., 2009).

### Central Sensitization

Central sensitization involves the increased responsiveness of nociceptive neurons in the CNS that can be generated from intense and repeated peripheral inputs. Sensitized C-fibers lead to the increased release of neurotransmitters such as glutamate, substance P (SP), calcitonin gene-related peptide (CGRP), and brain-derived neurotrophic factor (BDNF) into central terminals of the spinal cord dorsal horn (postsynaptic). Released neurotransmitters bind to their receptors, including *N*-methyl-D-aspartate receptors (NMDAR), α-Amino-3-hydroxy-5-methyl-4-isoxazolepropionic acid receptors (AMPAR), metabotropic glutamate receptors (mGluR), neurokinin-1 receptors (NK-1R), and tropomyosin receptor kinase (Trk) receptors, on the postsynaptic neurons. These series of processes induce hyperactive/hyperexcited states in the postsynaptic neurons and result in hyperalgesia (an abnormal increase in pain sensitivity from painful stimuli), allodynia (pain caused by non-painful stimuli), and the expansion of the receptive fields of nociceptors (Hylden et al., 1989; Latremoliere and Woolf, 2009; Woolf, 2011). Additionally, peripheral injury can redistribute the central terminals of myelinated afferents and induce pain by non-painful afferents (Woolf et al., 1992; Tashima et al., 2018; Nagi et al., 2019). This process seems similar to peripheral sensitization in so far as it also results in hyperalgesia and allodynia but differs in the range of sites that cause the resulting pain. Peripheral sensitization occurs when nociceptors are exposed to inflammatory mediators and damaged tissue. Consequently, the abnormal pain sensation prompted by peripheral sensitization is limited to inflamed or damaged sites. Hence, this phenomenon can function to protect sites of injury. By contrast, central sensitization can induce pain either in sites where inflammation or injury has already been resolved or without no obvious inflammation or injury.

Intracellular Ca^2+^ is important to initiating central sensitization. Under normal conditions, the pores of NMDARs are blocked by Mg^2+^. However, glutamates released from presynaptic buds dislodge Mg^2+^ and allow Ca^2+^ to enter the cytosol. Further, activation of the group I mGluRs (mGluR1 and 5) opens the Ca^2+^ channels on the endoplasmic reticulum (ER). Intracellular Ca^2+^ activates protein kinase C (PKC), PKA, and calmodulin-dependent protein kinase II (CaMKII). These kinases phosphorylate ion channels and glutamate receptors, and their properties are rapidly changed. PKC phosphorylates Ser831 on the GluR1 of AMPAR, Ser880 on GluR2, and Ser896 on NR1 of the NMDAR subunit. PKA phosphorylates Ser890 and Ser897 on NR1 and Ser845 on GluR1. CaMKII phosphorylates Ser1303 on NR2B and Ser831 on GluR1 (Suo et al., 2013; Ji et al., 2018). These processes correspond to the early, phosphorylation-dependent phase of central sensitization in which extracellular signal-regulated protein kinase (ERK) is important to sustain central sensitization. The activation of ERK stimulates increases in the transcription of several genes, such as c-*Fos*, NK1, TrkB, and Cox-2, the phosphorylation of NMDAR and Ser616 on Kv4.2, and the insertion of AMPAR into the membrane. Together, these events increase neuronal excitability in the spinal cord (Ji and Woolf, 2001; Pace et al., 2018).

## TRP Channel and Pain

Unpleasant and painful sensations signal organisms to subsequently avoid these situations, allowing the organisms to survive noxious insults and injuries. Such noxious stimuli are detected by nociceptors, specialized peripheral sensory neurons that send the threatening signals to the spinal cord and the brain. If a stimulus is intense enough to reach the noxious range, ion channels expressed in the nociceptors are activated and generate action potentials. Ion channels called TRP channels are considered to play an important role in transducing noxious thermal, chemical, and mechanical stimuli.

The TRP channels were first discovered in the eyes of a TRP-mutant *Drosophila melanogaster*, where the mutant fly displayed a transient abnormal response to a bright light, while wild type flies showed a sustained response (Cosens and Manning, 1969). Later, TRP channels in mammalian were introduced with an extended protein family comprising more than 30 distinct subtypes (Ramsey et al., 2006). The TRP family can be distinguished into 6 members: TRPV (vanilloid), TRPA (ankyrin), TRPM (melastatin), TRPC (canonical), TRPP (polycystin), and TRPML (mucolipin) (Chung and Caterina, 2007). These TRP families are similar in architecture, having six putative transmembrane domains with cytoplasmic amino and carboxyl termini, although they differ in amino-acid conservation. Among these members of TRP family, at least six subtypes, including TRPV1-4, TRPA1, and TRPM8, are closely related to painful thermal, chemical, and mechanical stimuli evoking nociceptive pain (Patapoutian et al., 2009; Dai, 2016). Intracellular Ca^2+^ concentration increases in response to stimuli that are specific to certain TRP channels, such as Ca^2+^ entry channels (Zheng, 2013). TRP channels also stimulate intracellular signaling pathways (e.g., PKC activity) and cause transcriptional changes, all of which promote the expression and release of pro-inflammatory peptides such as SP and CGRP (Veldhuis et al., 2015). Moreover, G protein-coupled receptors (GPCRs) are located in the plasma membrane along with TRP channels and are responsible for the central transduction of painful signals from the periphery nociceptors. These GPCRs are activated by noxious stimuli from the extracellular environment and control TRP channel activity through several mechanisms, wherein the G-protein subunits stimulate second messenger kinases [e.g., cyclic adenosine monophosphate (cAMP)-dependent protein kinase A, phospholipase C (PLC), PKC] (Veldhuis et al., 2015).

### TRPV1 as Thermal Sensor

Transient receptor potential channels specifically related to pain and thermosensation were first suggested when the capsaicin receptor, TRPV1, was cloned from a rodent dorsal root ganglia (DRG) in 1997 and was reported as a non-selective cation channel exhibiting high calcium permeability (Caterina, 2007). TRPV1 is mainly expressed in small diameter neurons in the DRG and trigeminal ganglion (TG). TRPV1 is activated by both noxious heat (≥43°C) and capsaicin, the pungent ingredient of chili peppers, triggering a sensation of burning pain. In addition to capsaicin and noxious heat, TRPV1 is also activated by spider toxin (Siemens et al., 2006) and low pH (protons) (Caterina et al., 1997). Therefore, TRPV1 is defined as a polymodal receptor (Clapham, 2003). Moreover, certain studies revealed that TRPV1-deficient mice had a complete loss of physiological and behavioral responses to capsaicin, along with significant impairment in responses to noxious heat and mechanical stimuli (Caterina et al., 2000; Davis et al., 2000; Bolcskei et al., 2005). However, other studies reported that TRPV1 null mice had no differences in sensitivity to acute noxious heat compared to wild type mice (Davis et al., 2000; Woodbury et al., 2004). This suggests that TRPV1 is not solely responsible for the sensation of noxious heat, indicating the presence of other possible compensatory mechanisms.

### TRPA1 as a Chemical and Thermal Sensor

Another non-selective cation channel of the TRP family subtypes, TRPA1, is also found to be critical in nociception. TRPA1 is highly co-expressed with TRPV1 in small-diameter nociceptors in the DRG and TG and can be influenced by TRPV1 (Salas et al., 2009; Staruschenko et al., 2010). TRPA1 is a polymodal receptor, like TRPV1 (Story et al., 2003; Dai et al., 2007). TRPA1 is activated by a variety of noxious stimuli that induce acute painful burning or stinging sensations including chemicals, such as mustard oil (isothiocyanates), garlic (allicin), and cinnamon oil (cinnamaldehyde) (Bandell et al., 2004; Jordt et al., 2004; Bautista et al., 2005; Macpherson et al., 2005). These chemicals are all electrophilic and activate TRPA1 by covalent modification of cysteine residues in the channel, suggesting that the reactivity of such chemicals is not necessarily dependent on its structure (Hinman et al., 2006; Macpherson et al., 2007). In TRPA1 knockout mice studies, those chemicals showed reduced nociceptive pain behavior (Macpherson et al., 2007; McNamara et al., 2007), indicating TRPA1 as a key detector of chemical damage. Additionally, TRPA1 has also been suggested to detect noxious cold stimuli (<15°C) (Story et al., 2003; Sawada et al., 2008), even though studies of TRPA1-deficient mice showed discordant results (Bandell et al., 2004; Jordt et al., 2004; Zurborg et al., 2007; Karashima et al., 2009).

## TRP Channels in Inflammatory Pain Conditions

Depending on pathological conditions such as inflammation (inflammatory pain) or nerve injury (neuropathic pain), changes occur in the expression of TRP channels and their function, including transcriptional and translational regulation and post-translational alteration (Patapoutian et al., 2009). As mentioned above, inflammatory pain is generated from the activation and sensitization of nociceptive pain signaling in the form of a reduced threshold and an increased responsiveness of nociceptors. Three cellular and molecular mechanisms have been suggested in the development of inflammatory pain involved in TRP channels. First, TRP channel expression increases in the sensory neurons either transcriptionally or post-translationally during inflammation. For instance, the activation of C-C chemokine receptor type 2 (CCR2) by the macrophage inflammatory protein-1α (MIP-1α/CCL3) increases the transcription of TRPV1 and TRPA1 (Jung et al., 2008); i.e., TRPV1 levels in peripheral terminal or nociceptors are increased in order to maintain the inflammatory heat hypersensitivity (Ji et al., 2002). TRPA1 in DRG neurons is also upregulated by peripheral inflammation transcriptionally, leading to inflammatory cold hyperalgesia (Obata et al., 2005). In other words, the increase in expression and the oxidative products from tissue damage and inflammation led to a drastic decrease in the activation threshold, thereby increasing sensitivity to noxious stimuli (via peripheral sensitization) (Hucho and Levine, 2007). Second, translocation of functional TRP channels from the cytoplasm to the plasma membrane is induced following the activation of second-messenger pathways and subsequent post-translational modification (e.g., channel phosphorylation or glycosylation) (Morenilla-Palao et al., 2004; Nguyen et al., 2005; Zhang et al., 2005). Third, channel phosphorylation as a disinhibition mechanism with inflammatory mediators might cause channel structure alteration and functionally enhance the channel sensitivity (Dai et al., 2007). Tumor necrosis factor (TNF)-α increases the frequency, not the amplitude, of spontaneous excitatory postsynaptic currents (sEPSCs) in wild type mice but not in TRPV1 KO mice (Park et al., 2011a). Importantly, studies conducted using knockout mice lacking TRPV1 showed that the development of inflammatory thermal hyperalgesia became defective (Keeble et al., 2005; Barton et al., 2006). This suggests that TRPV1 is a key component of the mechanism in which inflammation causes thermal hyperalgesia and pain hypersensitivity (Caterina et al., 2000; Davis et al., 2000; Bolcskei et al., 2005; Basbaum et al., 2009). In addition to TRPV1, TRPA1-deficient mice studies also showed markedly reduced development of hyperalgesia in response to inflammation-related chemicals that were injected such as formalin and bradykinin (Bautista et al., 2006; McNamara et al., 2007; Andersson et al., 2008).

## TRP Channels in Neuropathic Pain Conditions

Physiological changes such as increasing the local Ca^2+^ ion concentration in primary sensory neurons or in the spinal cord owing to nerve injury consequently affect signal processing in the CNS, evoking neuropathic pain (Fernyhough and Calcutt, 2010). When neuropathic pain impulses are transmitted from the periphery to the CNS, nociceptive transmitters such as SP are released by exocytosis from the primary sensory terminals in the spinal dorsal horn, where TRP channels are mediated by voltage-dependent Ca^2+^ channels (Verkhratsky and Fernyhough, 2008).

After nerve damage, expression in TRP channel changes dynamically in sensory neurons, and different TRP channels are involved in the management of neuropathic pain. In the spinal nerve section model, the expression of TRPV1 mRNA is reduced in the axotomized ganglia, suggesting loss of trophic support after the injury (Michael and Priestley, 1999). In L5 spinal nerve ligation model, TRPV1, TRPA1, and TRPM8 are upregulated in uninjured L4 somata (Hudson et al., 2001; Obata et al., 2005). It has been suggested that the injured neurons might release growth factors and neurotransmitters into the surrounding region, causing an increase in the excitability of nearby uninjured spared neurons (Fukuoka et al., 2001; Sexton et al., 2014). Vilceanu et al. (2010) revealed that the proportion of TRPV1-expressing DRG neurons was increased and its TRPV1 function was improved after nerve injury induced by spinal nerve ligation (SNL). Moreover, sciatic nerve transection in rats results in up-regulation of TRPV1 at the central terminals of the primary afferent neurons in the spinal cord, increased release of inflammatory neuropeptides such as calcitonin gene-related peptide and SP from the presynaptic central terminals, and enhanced glutamatergic neurotransmission (Kanai et al., 2005; Lappin et al., 2006; Lee and Kim, 2007; Spicarova et al., 2011). Both TRPV1 and TRPA1 are involved in peripheral neuropathy and neuropathic pain induced by chemotherapeutic agents such as cisplatin, oxaliplatin, and paclitaxel. Inhibition of TRPA1 function eliminates mechanical as well as cold allodynia induced by cisplatin and oxaliplatin, which are most commonly used for chemotherapy (Baron, 2009; Nassini et al., 2011; Zhao et al., 2012). Paclitaxel chemotherapy mediates neuropathic pain behaviors by the release of mast cell tryptase to activate the protease-activated receptor 2 (PAR 2), which then sensitizes TRPV1, TRPV4, and TRPA1 via PLC, PKC, and PKA signaling (Chen et al., 2011); it also enhances the TRPV1 mRNA transcripts and TRPV1 protein in small-to-medium diameter DRG neurons (Hara et al., 2013). Additionally, in neuropathic pain induced by a chronic constriction injury model, inhibition of TRPA1 function also diminishes cold allodynia (Hara et al., 2013).

**FIGURE 1:**
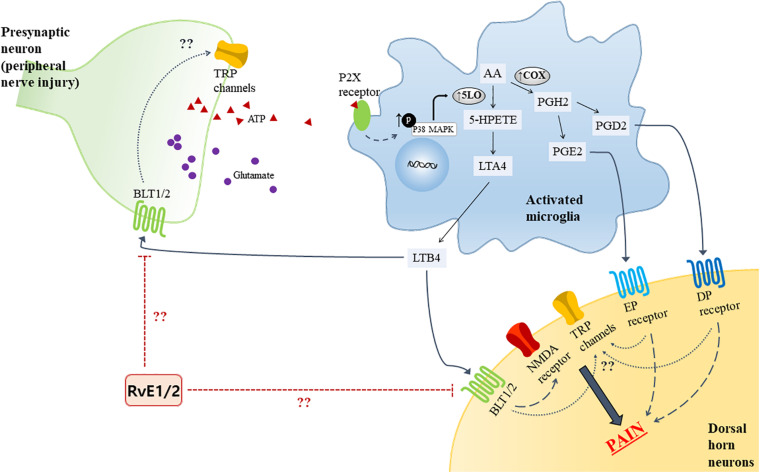
Proposed model for LTs and PGs synthase mechanism from AA in activated microglia by ATP due to peripheral nerve injury, and the receptors in the spinal cord. The activated microglia phosphorylate p38 MAPK, upregulating 5-LO expression. Bioactive LTB4, PGE2, and PGD2 bind to their receptors (BLT1/2, EP and DP receptors, respectively) and evoke pain. The possible analgesic role of RvE1 and E2, which is known to be antagonists of BLT1, and the relationship TRP channel in the presynaptic and spinal dorsal horn neuron are depicted. AA, Arachidonic acid; ATP, adenosine triphosphate; BLT, leukotriene B4 receptor; DP, D–type prostanoid; EP, E-type prostanoid; MAPK, mitogen-activated protein kinases; PG, prostaglandin; PGD, prostaglandin D; PGE, prostaglandin E; RvE1, resolvin E1; RvE2, resolving E2; TRP, transient receptor potential; 5-LO, 5-lipoxygenase.

Certain pro-inflammatory lipid mediators [e.g., leukotrienes (LTs) and prostaglandins (PGs)] in the spinal cord reportedly contribute to neuropathic pain. Of interest, a growing evidence has established that microglial activation has emerged as key players in maintaining pain hypersensitivity in the dorsal horn. Together, the activation of microglia induces dramatic changes including an intracellular signaling molecules [e.g., mitogen-activated protein kinases (MAPKs)], leading to increase the production of pro-inflammatory lipid mediators in the microglia. Following peripheral nerve injury, adenosine triphosphate (ATP) is known to be released from the primary afferent central terminals in the spinal cord, and studies have shown that expression of the purinergic P2 (P2X) receptors was upregulated in microglia to be able to detect extracellular ATP in the spinal cord (Tsuda et al., 2003; Ulmann et al., 2008). LTs are a group of lipid mediators derived from arachidonic acid (AA) which is converted into leukotriene A4 (LTA4) via the 5-lipoxygenase (5-LO) pathway in microglia. LTA4 is then enzymatically converted into LTB4 (Noguchi and Okubo, 2011). In the rat model of spared nerve injury (SNI), LTB4 was expressed in both the spinal neurons and microglia along with LTB4 receptor 1 (BLT1), and the BLT1 was expressed in the spinal neurons. The effect of LTs on neuropathic pain behaviors was identified by intrathecal administration of a 5-LO inhibitor that attenuated the mechanical hypersensitivity caused by SNI surgery (Okubo et al., 2010). Okubo and researchers also found that the increase of 5-LO in spinal microglia was reduced on treatment with an p38 MAPK inhibitor, but not mitogen-activated protein kinase (MEK) inhibitor, indicating that the p38 MAPK pathway is crucial in the generation of neuropathic pain (Figure 1). While BLT1 binds LTB4 as its agonist, resolvin E1 and E2 are considered as endogenous receptor antagonists for BLT1. A previous study showed that intrathecal pre-treatment of RvE1 attenuated neuropathic pain by modulating microglial activation in the spinal cord (Xu et al., 2013); thus, there may be an association of BLT1 with RvE1 and E2. In the context of AA metabolites, it is also known to be metabolized into the epoxygenase metabolite, 5,6-EET, which directly activates TRPV4 channels (Vriens et al., 2004). However, not all AA-derived mediators have pro-inflammatory action. Lipoxin A4 (LXA4) is an eicosanoid derived from AA through sequential actions of lipoxygenases, 15-LO and 5-LO; it is known to be an agonist for ALX/FPR2, acting as an endogenous “stop” signal in acute inflammation to switch into the resolution phase (Levy et al., 2001). Martini et al. showed that treatment with LXA4 modulated microglial activation and TNF-α release via ALX/FPR2 receptors, thereby reducing neuropathic pain in rodents after spinal cord hemisection (Martini et al., 2016). PG, a pro-inflammatory mediator synthesized from AA by the COX enzyme in microglia, has been identified as another factor in mechanical allodynia in an SNI model. PGH2 is synthesized from AA by the action of COX and serves as a substrate of the prostaglandin synthase enzymes for producing the bioactive prostaglandins, PGE2 and PGD2, which bind to the EP and DP receptors, respectively (Kanda et al., 2013). Like LTB4, p38 activation in spinal microglia induces the release of PGE2 (Ji and Suter, 2007). Nakayama et al. reported an increase in PGE2 concentration and activation of EP1 receptors in the spinal dorsal horn in the late phase after carrageenan-induced mechanical hyperalgesia in rats (Nakayama et al., 2002). Moreover, following peripheral injury, an increase in PGD2 was noted, which was subsequent to the upregulation of COX-1, and the intrathecal injection of DP2 receptor antagonists attenuated the mechanical allodynia (Kanda et al., 2013). The activation of both EP and DP receptor initiate G-protein coupling to increase intracellular concentration of Ca^2+^ or cAMP in the dorsal horn (Jang et al., 2020). Although evidence of the mechanisms of prostaglandin and its receptors have been accumulated, their role in neuropathic pain and the resolution strategy remain unknown.

Moreover, these pathways of pro-inflammatory lipid mediators contributing to neuropathic pathways has not been studied in the context of TRP regulation. Moreover, even though these findings do not involve TRP regulation, LTs and PGs might be implicated in neuropathic pain by acting like G-protein-mediated signal transducers to initiate second messenger systems, which in turn activate TRP channels (Figure 1). Interestingly, TRPV1 is found to be upregulated in the spinal cord dorsal horn in the chronic constriction injury (CCI) neuropathic pain model, indicating possible interaction between the receptors mentioned above (Kanai et al., 2005). Further research will contribute identifying the analgesic effect caused by those pro-inflammatory mediators on neuropathic pain.

Transient receptor potential channels are upregulated in order to manage hypersensitivity of both inflammatory and neuropathic pain at the site of inflammation and the remaining intact neurons close to the damaged nerve, respectively. However, different mediators and intracellular mechanisms of TRP channels are involved, depending on what type of pain is occurring. Hence, it is important to elaborate the role of TRP channels in the resolution of different types of pathological pain using resolvin, which will be discussed in detail next.

## Resolvins (Endogenous Lipid Mediators) Inhibit TRP Channels

### Lipid Mediators in Resolution of Acute Inflammation

Acute inflammation is a host defense system, and its resolution may serve as a gateway to chronic inflammation. In health status, inflammatory responses are self-limited, with many cell types and tissues involved in initiation and termination of acute inflammation (Serhan, 2007). Tissue injury or infection triggers the release of a train of signals, including damage-associated molecular patterns (DAMPs), pathogen-associated molecular patterns (PAMPs), lipid mediators and chemokines, such as formylmethionyl-leucyl-phenylalanine (fMLP), ATP, high mobility group box-1 (HMGB1), F-actin, N-glycan, LTB4, CCR1 ligands, chemokine (C-X-C motif) ligand (CXCL) 2, and CXCL8, from injury sites (McDonald and Kubes, 2010; Selders et al., 2017; Peiseler and Kubes, 2019; Gong et al., 2020). Neutrophils move through the chemoattractant gradient, while the integrin molecules on neutrophil surface bind to integrin receptors on endothelial cells enabling their infiltration into the tissues. The infiltrated neutrophils remove pathogens via phagocytosis and release reactive oxygen species (ROS), degradative enzymes, microbicidal agents, and neutrophil extracellular traps (Metzemaekers et al., 2020). Neutrophils are the first defender against hazardous invaders, but their high numbers can induce excessive inflammation (Freire and Van Dyke, 2013). Since excessive inflammation is considered to be a component in many chronic diseases, such as vascular diseases, metabolic syndrome, neurological diseases, and many others, proper resolution of acute inflammation is important. (Serhan et al., 2015b). Resolution of acute inflammation was introduced as a passive process following disappearance of the chemoattractant and other chemical mediators, but Dr. Serhan’s group uncovered endogenous lipid mediators in inflammatory exudate: resolvins, protectins, and maresins (Serhan et al., 2002; Serhan, 2014). These pro-resolving lipid mediators are often referred to as specialized pro-resolving mediators (SPMs) because they stimulate efferocytosis of polymorphonuclear leukocytes (PMNs) to facilitate complete removal of pathogens, recruit macrophages, attenuate activated neutrophil through NF-κB inhibition, and initiate the resolution phase of acute inflammation (Flower and Perretti, 2005; Lee et al., 2013; Francos-Quijorna et al., 2017; Yurdagul et al., 2017; Rymut et al., 2020).

### Biosynthesis of Resolvins

Notable omega-3 fatty acids are alpha-linolenic acid (ALA), EPA, and DHA. In humans, ALA converts to EPA and DHA, but the conversion rate of ALA to EPA is 8–20%, and that to DHA is 0.5–9%, which are very low (Stark et al., 2008).

**FIGURE 2:**
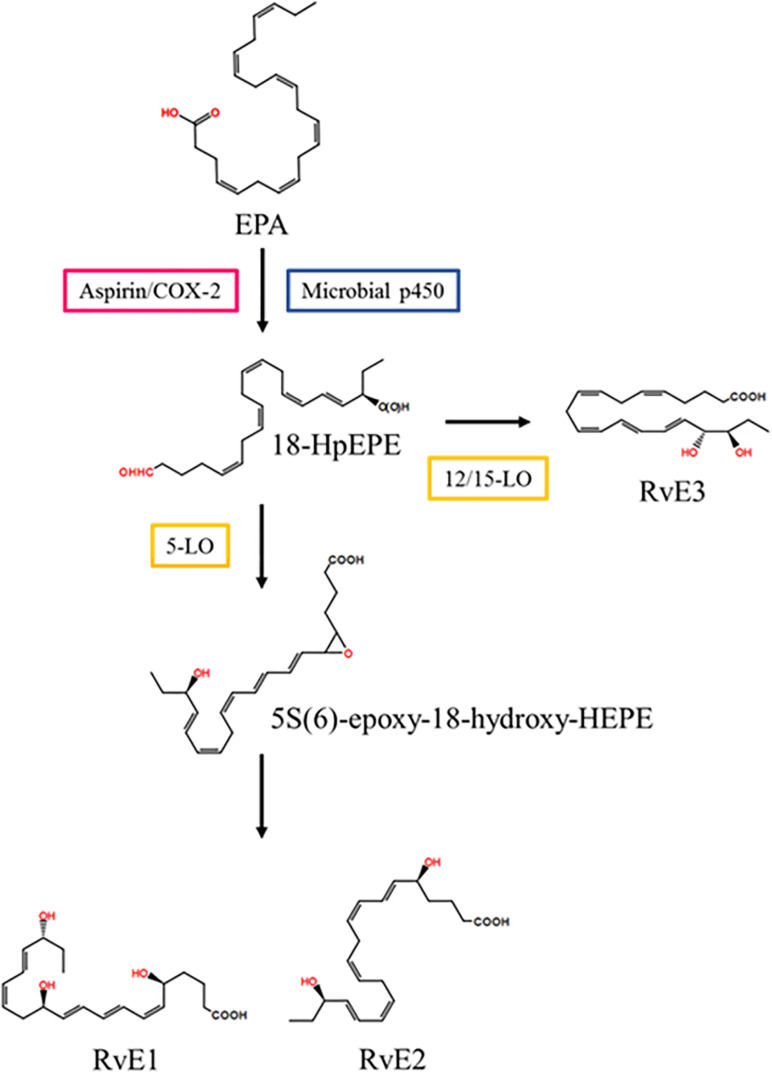
Resolvin E series biosynthetic pathway. EPA is converted into 18-HpEPE. 5S(6)-epoxy-18-hydroxy-HEPE is synthesized from 18-HpEPE with 5-LO and is transformed into RvD1 and RvD2. 18-HpEPE is also transformed into RvD3 by 12/15-LO. EPA, eicosapentaenoic acid; LO, lipoxygenase; RvD, resolvin D; 18-HpEPE, 18R-hydroperoxy-EPE.

In vascular endothelial cells, EPA is converted into 18R-hydroperoxy-EPE (18-HpEPE) with aspirin/COX-2 or microbial p450.18-HpEPE is converted to 5S(6)-epoxy-18-hydroxy-HEPE with 5- LO in leukocytes and then converted to resolvin E1 via enzymatic hydrolysis or to resolvin E2 via reduction (Serhan et al., 2015a). In eosinophils, 18-HpEPE is also converted to resolvin E3 via 12/15-LO pathways (Isobe et al., 2012) (Figure 2).

**FIGURE 3:**
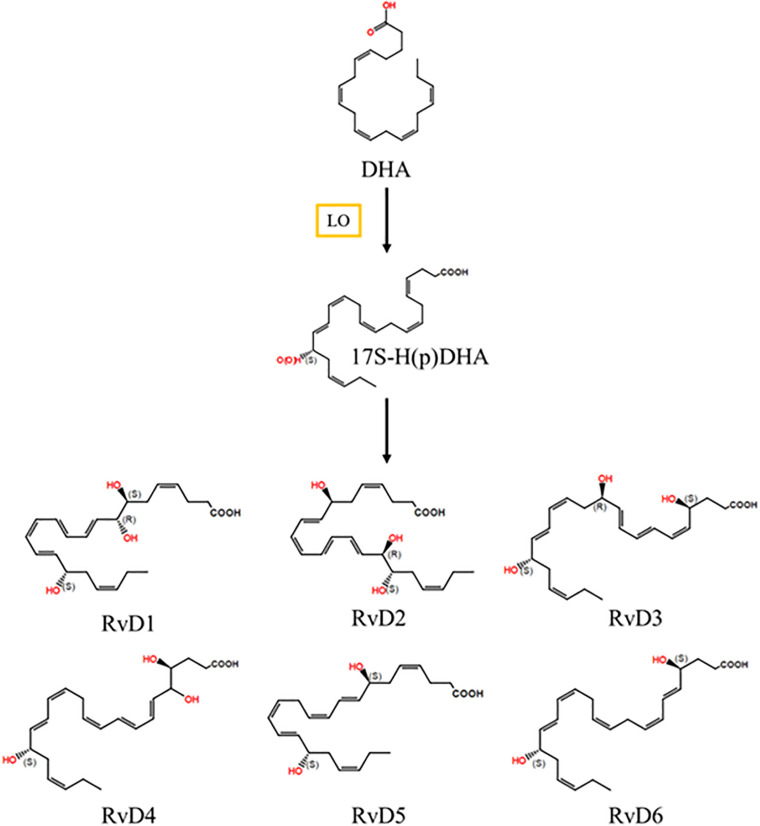
Resolvin D series biosynthetic pathway. DHA is converted into 17S-HpDHA by LO. 17S-HpDHA is further transformed into RvD1, RvD2, RvD3, RvD4, RvD5, and RvD6. DHA, docosahexaenoic acid; LO, lipoxygenase; RvD, resolvin D; 17-hydroperoxydocosahexaenoic acid.

In exudates, resolvin D series are biosynthesized by PMNs or macrophages. DHA is metabolized to 17-hydroxydocosahexaenoic acid (17-HDHA) via 17-hydroperoxydocosahexaenoic acid through 15-LO (Gleissman et al., 2010). 17-HDHA is converted to resolvin D1, D2, D3, D4, D5, and D6 through epoxidation and 5-LO (Figure 3). DHA is also metabolized with aspirin/COX-2, and then the aspirin-triggered resolvin D (AT-RvD) series is formed (Lim et al., 2015; Serhan et al., 2015a; Serhan and Levy, 2018). AT-RvD3 is more potent than resolvin D3 in phagocytosis of 10 and 100 pM zymosan (Dalli et al., 2013).

Since TRP channels are expressed in nociceptors where pain is generated, the roles of TRP channels in mediating pathological pain make them potential therapeutic targets for resolution. Until now, in addition to RvE1 and RvD1/AT-RvD1, more resolving family members have been biosynthesized: RvD2, RvD3, RvD4, and RvD5. RvE1, RvD1, and AT-TvD1 are already well-known agonists that resolve inflammation and inflammation-associated pain by mediating specific GPCRs [e.g., chemerin receptor23 (ChemR23) and GPR32], which are expressed by immune cells, glia cells, and neurons. This, in turn, reduces inflammation, glial activation, and spinal cord synaptic plasticity (Ji et al., 2011). Mounting evidence on resolvins as potent inhibitors for both inflammatory and neuropathic pain will be discussed with recent findings.

### Resolvin Receptors

**FIGURE 4:**
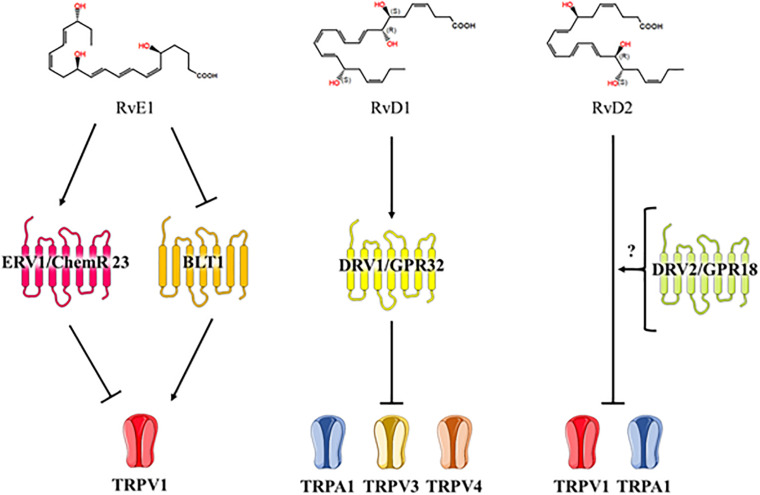
Resolvin series inhibit TRP channels. RvD, resolvin D; TRP, transient receptor potential.

Resolvins can directly act on nociceptors but little is known about how resolvins inhibit TRP channels in neurons. Five GPCRs – ERV/ChemR23, BLT1, ALX/FPR2, DRV1/GPR32, and DRV2/GPR18 – are receptors for resolvin D and E series that play an important role in the modulation of the TRP channels (Pirault and Back, 2018) (Figure 4).

ChemR23, resolvin E1 and chemerin receptors (Herova et al., 2015) are co-expressed with TRPV1 in small sized-DRG neurons and in the spinal cord dorsal horn. Activation of chemR23 by resolvin E1 or chemerin blocked capsaicin-evoked spontaneous excitatory postsynaptic current frequency increases via the ERK pathway in the spinal cord dorsal horn, and resolvin E1 also blocked phosphorylation of ERK by capsaicin treatment in DRG neurons (Xu et al., 2010; Park et al., 2011b; Jo et al., 2016). ERK is a member of the mitogen-activated protein kinase superfamily of signaling pathways, an important kinase in painful conditions. In addition to capsaicin, noxious heat, cold, and prick upregulate the pERK intensity in neurons (Ji et al., 1999). In particular, pERK is upregulated in DRG and in the spinal cord in neuropathic pain models of partial sciatic nerve ligation (PSNL), SNL, CCI and diabetes (Seltzer et al., 1990; Song et al., 2005; Liu et al., 2012; Xu et al., 2014; Guo et al., 2019; Wang et al., 2020).

BLT1, LTB4 (agonist), and resolvin E1 (antagonist) receptor are co-expressed with TRPV1 in DRG neurons (Andoh and Kuraishi, 2005; Serhan and Chiang, 2013). LTB4 functions as an agonist and sensitizes TRPV1-mediated Ca^2+^ increase. Although pain behavior in the second phase of the formalin pain model is significantly attenuated in BLT1-KO mice relative to WT mice, no analgesic effect has been observed in the first phase. This is because the first phase is mainly mediated by *TRPA1* and *TRPV1* gene expression, which do not change in the DRG and spinal cords of BLT1-KO mice. Hence, BLT1 may not be involved in mediating the properties of TRPA1 (McNamara et al., 2007; Asahara et al., 2015).

Resolvin D1 inhibits TRPA1, TRPV3, and TRPV4 in heterogeneous systems. FPR2/ALX is a resolvin D1 receptor, but treatment with FPR2/ALX agonists, cathelicidin LL-37 and Trp-Lys-Tyr-Met-Val-Met (WKYMVM), do not affect TRPA1, TPRV3, and TRPV4 inhibition (Bang et al., 2010). However, there is another resolvin D1 receptor, GPR32, that is also activated by AT-RvD1. FPR2/ALX and GPR32 are GPCRs that regulate specific microRNAs and their target genes that promote the resolution of acute inflammation (Krishnamoorthy et al., 2012).

Resolvin D2 is known to activate a cell surface GPCR, GPR18/DRV2 (Chiang et al., 2015, 2017). GPR18 is expressed in lumbar DRG and in the spinal cord at a genetic level and under neuropathic conditions; it is also upregulated in the spinal cord (Malek et al., 2016). Resolvin D2 inhibits TRPV1 and TRPA1 activation dose-dependently. Resolvin D2 is ten times more potent than resolvin E1 on TRPV1 inhibition (IC_50_ = 0.04 ± 0.01 and 0.4 ± 0.05, respectively), and it is four times more potent than resolvin D1 on TRPA1 inhibition (IC_50_ = 0.8 ± 0.2 and 3.2 ± 0.05, respectively) (Park et al., 2011b).

## Resolvins Alleviate Chronic Pain

### Resolvins in the Resolution of Inflammatory Pain

Studies have shown that both peripheral (intraplanar) and central (intrathecal) administration of RvE1 and RvD1 reduce inflammatory pain (Bang et al., 2010; Xu et al., 2010; Lima-Garcia et al., 2011; Park et al., 2011b) and postoperative pain (Huang et al., 2011; Wang and Strichartz, 2017) by modulating the activation of TRP channels. Bang et al. reported that pretreatment with RvD1 (20 ng) suppressed pain behaviors induced by intraplanar formalin injection (Bang et al., 2010) and attempted to determine whether activators of the RvD1 receptor (FPR2/ALX) inhibits the effect of TRP channels (TRPV3/4 and A1), however, no such effect was found (Krishnamoorthy et al., 2010). Xu et al. (2010) found that both RvD1 and RvE1 (1–20 ng) suppressed inflammatory pain behaviors induced by formalin, carrageenan, or complete Freund’s adjuvant (CFA); they also indicated that the possible mechanism where RvE1 inhibits ERK signaling pathways to block TNF-α induced increased EPSC frequency and NMDAR hyperactivity in spinal dorsal horn neurons. Additionally, an agonist of ChemR23, which was considered to be an RvE1 receptor, also abolished capsaicin-induced increase in sEPSC frequency, implicating the role of RvE1 in mediating TRPV1 activity via ChemR23. Moreover, Park et al. examined RvD2 at very low doses (10 ng) injected intrathecally and found that it prevented spontaneous pain induced by formalin as well as reversed inflammatory pain induced by CFA (Park et al., 2011b). They identified RvD2 as endogenous inhibitors both for TRPV1 and TRPA1, by which RvD2 acts on specific GPCRs. Table 1 shows a list of the published findings of the resolution effect of resolvins specifically on TRP channels for inflammatory pain.

**TABLE 1:** Analgesic effects of different resolvins in distinctive TRP channels for inflammatory and neuropathic pain.

Research article	Pain type	Resolvin	TRP channel	Results	Potential mechanisms
Bang et al., 2010	Inflammatory	RvD1	TRPA1 TRPV3 TRPV4	↓activities the three TRP channels at nanomolar and micromolar levels **No effects of FPR2/ALX agonists on the three TRP channels	
Xu et al., 2010	Inflammatory	RvE1 RvD1	TRPV1	↓EPSC frequency increases induced by capsaicin, with the ChemR23 agonist ↓EPSC frequency increase by TNF-α and NMDAR hyperactivity with RvE1	RvE1 modulates the ERK signaling pathway to abolish TNF-α-evoked NMDA receptor hyperactivity in dorsal horn neurons
Park et al., 2011b	Inflammatory	RvD1 RvD2 RvE1	TRPV1 TRPA1	↓sEPSC increases both TRP channels with RvD2 at extremely low doses (compared to RvD1/E1) **No effect of RvD2 when GPCRs are blocked	Distinct mechanisms of the resolvins in regulating TRP channels RvD2 involves specific GPCRs
Luo et al., 2019	Neuropathic (CIPN induced) Inflammatory (**RvD5 only)	RvD1 RvD2 RvD5	TRPA1 TRPV1 (**Knock-out mice)	↓ neuropathic pain behaviors, with RvD1/D2 ** *only in male mice, with RvD5* ↓inflammatory pain behaviors in male mice only with RvD5	Sex dimorphism of RvD5’s analgesia in both pain models

CFA, complete Freund’s adjuvant; CIPN, chemotherapy-induced peripheral neuropathy; EPSC, excitatory postsynaptic current; ERK, extracellular signal-regulated kinase; FPR2/ALX, formyl peptide receptor 2; GPCRs, G-protein coupled receptors; NMDAR, *N*-methyl-D-aspartic acid receptor; RvE, resolvin E; RvD, resolvin D; TNF-α, tumor necrosis factor-α; TRP, transient receptor potential. ** Indicates supplementary information.

### Resolvins in the Resolution of Neuropathic Pain

While there is mounting research on the analgesic effect of resolvins in inflammatory pain, the role of resolvins in TRP channels to alleviate neuropathic pain is not well known. One study evaluated the analgesic actions of resolvin D-series (D1 to D5) in a TRPA1 and TRPV1 knockout mouse model of chemotherapy-induced peripheral neuropathy induced by paclitaxel (Luo et al., 2019). They found that RvD1 and RvD2 reduced mechanical allodynia in both sexes of TRPV1 and TRPA1 deficient mice, whereas RvD3 and RvD4 had no effects on either sex. Interestingly, RvD5 reduced mechanical allodynia only in male mice but not in female mice, indicating there is a sex dimorphism in pain regulation. Since RvD5 affected the TRPV1 and TRPA1 knockout male mice only, this might implicate the TRP channel in the role of RvD5 to attenuate neuropathic pain in female mice.

## Conclusion: Importance of Pain Relief Using Resolvins via TRP Modulation

Opioid and non-opioid analgesic drugs for the treatment acute and chronic pain. Opioids are a type of medications that are used to relieve severe chronic pain. However, as opioid receptors are expressed throughout the entire nervous system and gastrointestinal tract and do not operate on specific pain pathways, their abuse and misuse can induce unexpected physiological and psychological side effects that devastate the lives of the affected individuals and their families. Morphine, one of the most widely prescribed opioid analgesics, increases pain hypersensitivity at low doses (Ghelardini et al., 2015). Morphine-6-glucuronide (M6G), a metabolite of morphine, has more potent analgesic effect than does morphine. However, M6G is dangerous to patients with renal failure and relate to respiratory depression (Lotsch, 2005). For these reasons, several nations and international organizations have thoroughly controlled the prescription and use of opioids (Rosenblum et al., 2008; Rogers et al., 2013). Further, the consequences of opioid use underscore the need to develop non-opioid analgesics and thus minimize the prescription of opioid analgesics.

Non-opioid analgesics include both selective and non-selective cyclooxygenase (COX) inhibitors, as well as well-known non-steroidal anti-inflammatory drugs and acetaminophen. COX contributes to the synthesis of PG, which causes inflammation, pain and fever. Because of this, COX inhibitors are used for antipyretics and antiphlogistics, as well as analgesics (Qureshi and Dua, 2020). However, the administration of COX-2 inhibitors can induce cardiovascular complications (Nussmeier et al., 2005).

Therefore, it is necessary to develop new analgesic targets that are specific to nociceptive neurons. One of the targets is TRP channels that mediate nociception, especially TRPV1. Many models have shown that TRPV1 inhibition reduces chronic pain including inflammatory and neuropathic pain. TRP channels in nerve systems have been emerging targets for pain management, and several TRPV1 antagonists (ABT-102, AMG-517, AZD-1386, DWP-05195, JTS-653, MK-2295, PHE-377 and SB-705498) have already entered clinical trials (Wu et al., 2018). However, TRPV1 antagonists can induce abnormal changes in body temperature (hyperthermia and hypothermia (Gavva et al., 2008; Garami et al., 2018, 2020) or decrease heat sensation (Crutchlow et al., 2009). For these reasons, many TRPV1 antagonists have failed to be developed as painkillers.

Nevertheless, TRPV1 inhibitors are still being discovered. Resolvins, a class of lipid mediators, has been identified in this capacity. Resolvins are produced from omega-3 fatty acids and therefore have a great potential for reducing the incidence of side effects associated with pain management. Further, the fact that resolvins can attenuate powerful type cardiovascular disease enhances its potential as a useful analgesic (Salic et al., 2016; Capo et al., 2018). The analgesic role of resolvins has already been proven at nanomolar levels, resolvins are excellent potential therapeutic targets for pain. However, most current studies of resolvins in an analgesic role have been limited to inflammatory pain. Therefore, more studies on the analgesic effect of resolvins for neuropathic pain are needed.

## Author Contributions

C-KP and YHK conceived and supervised the project. JR, EJG, J-WP, YHK, and C-KP wrote the manuscript. All authors contributed to the article and approved the submitted version.

## Conflict of Interest

The authors declare that the research was conducted in the absence of any commercial or financial relationships that could be construed as a potential conflict of interest.
